# Extracellular vesicles as integrative hubs of drug resistance in bladder cancer

**DOI:** 10.20517/cdr.2026.118

**Published:** 2026-10-21

**Authors:** Cheng-Shuo Huang, Cheng-Ping Yu, Dah-Shyong Yu, Ying-Si Wu, Shih Sheng Jiang, Ruiying Zhao, Dung-Fang Lee, Jar-Yi Ho

**Affiliations:** ^1^Graduate Institute of Life Sciences, College of Biomedical Sciences, National Defense Medical University, Taipei 114, Taiwan.; ^2^Graduate Institute of Pathology and Parasitology, College of Medicine, National Defense Medical University, Taipei 114, Taiwan.; ^3^Department of Pathology, Tri-Service General Hospital, National Defense Medical University, Taipei 114, Taiwan.; ^4^National Institute of Cancer Research, National Health Research Institutes, Miaoli 35053, Taiwan.; ^5^Department of Integrative Biology and Pharmacology, McGovern Medical School, the University of Texas Health Science Center at Houston, Houston, TX 77030, USA.; ^6^Division of Urology, Department of Surgery, Tri-Service General Hospital, National Defense Medical University, Taipei 114, Taiwan.

**Keywords:** Extracellular vesicles, bladder cancer, drug resistance, gemcitabine resistance, platinum resistance, BCG failure, immunotherapy resistance, urinary extracellular vesicles

## Abstract

Therapeutic resistance remains a major challenge in bladder cancer, encompassing resistance to intravesical Bacillus Calmette–Guérin (BCG) therapy, platinum- and gemcitabine-based chemotherapy and immune-checkpoint blockade. Although extracellular vesicles (EVs) have been implicated in cancer drug resistance, their roles in bladder cancer are often discussed within the broader context of EV biology, making it difficult to distinguish direct mechanisms of treatment resistance from treatment-associated remodelling or biomarker associations. In this review, we refocus EV-mediated therapeutic resistance in bladder cancer and propose an evidence-stratified framework, distinguishing EVs as causal mediators, permissive amplifiers or biomarkers of drug adaptation. Studies have provided substantial mechanistic evidence from chemotherapy-associated models, including EV-mediated regulation of gemcitabine resistance through H3C14-associated EV remodelling and chemotherapy-exposed bladder cancer EVs that support stem-like, treatment-tolerant tumour states. Evidence supporting EV involvement in platinum resistance, BCG failure and immunotherapy resistance remains biologically plausible but less directly established. Therefore, clearer drug-specific models, improved EV-subtype resolution, cargo perturbation and functional rescue experiments are needed. We also discuss urinary EVs for longitudinal monitoring of treatment response and emerging resistance in bladder cancer. By integrating drug-specific therapeutic contexts with EV-specific causality criteria, this review provides a focused framework for evaluating when EVs mediate bladder cancer drug resistance, amplify adaptive tumour–microenvironment states or primarily serve as biomarkers for therapeutic monitoring.

## INTRODUCTION

Despite advances in multimodal therapy, acquired drug resistance and disease recurrence remain major obstacles in bladder cancer management. Non-muscle-invasive bladder cancer (NMIBC) is commonly treated with transurethral resection followed by intravesical therapy, including Bacillus Calmette–Guérin (BCG) therapy, whereas muscle-invasive cancer and advanced bladder cancer are frequently managed with multimodal treatment strategies, including surgery, platinum- and gemcitabine-based chemotherapy, immune-checkpoint blockade and other systemic approaches^[1-5]^. Although these treatments can achieve meaningful initial responses, durable disease control is often limited by BCG failure, chemotherapy resistance, immune escape and the emergence of treatment-adapted tumour cell states. BCG failure is particularly important in NMIBC, as a substantial proportion of patients fail to respond to treatment or relapse after treatment, and BCG-unresponsive disease represents a clinically defined state in which further BCG therapy is unlikely to provide benefit^[2,3,6]^.

Therapeutic failure has traditionally been attributed to cell-autonomous mechanisms driven by genetic alterations, clonal selection, altered drug transport, enhanced DNA damage repair and evasion of therapy-induced cell death. Nevertheless, this paradigm does not fully explain the dynamic, reversible and heterogeneous resistance phenotypes observed during bladder cancer progression. Accumulating evidence suggests that resistance arises through interactions among genetically distinct tumour clones, non-genetic cellular states and the tumour-microenvironment. Under drug-specific therapeutic pressure, bladder cancer cells can remodel signalling, metabolic, stem-like and survival programmes while reshaping immune, stromal and vascular compartments. In the context of BCG therapy, immune evasion may involve suppressive myeloid cells, tumour-associated macrophages, regulatory T cells, tolerogenic dendritic cells, exhausted effector T cells, cancer-associated fibroblasts (CAFs) and immune-checkpoint signalling^[7]^. Collectively, these reciprocal interactions establish adaptive tumour ecosystems that promote treatment tolerance without requiring every tumour cell to acquire a stable resistance phenotype^[8-12]^.

Within this adaptive landscape, extracellular vesicles (EVs) have emerged as important mediators of intercellular communication. By transferring proteins, nucleic acids, lipids and metabolites, EVs can mediate the exchange of molecular information among tumour, immune, stromal and endothelial compartments^[13-17]^. In bladder cancer, EV-mediated signalling is particularly relevant because EVs derived from both tumour and microenvironmental sources may influence drug response, immune modulation, phenotypic plasticity and treatment-associated adaptation. However, EVs are neither uniformly resistance-promoting nor the sole drivers of intercellular adaptation. Their biological effects depend on cellular origin, cargo composition, vesicle subtype, recipient-cell state and therapeutic context. Therefore, EV-associated findings should be interpreted in the context of specific bladder cancer therapies, including gemcitabine- and platinum-based chemotherapy, BCG therapy and immune-checkpoint blockade, rather than as general evidence of therapeutic resistance.

Recent studies illustrate how EVs may redistribute resistance-associated signals among bladder cancer cell populations. EV-mediated regulation of H3C14 has been implicated in gemcitabine resistance in a bladder cancer model, providing functional evidence that altered EV biology can influence sensitivity to gemcitabine^[18]^. Chemotherapy-exposed bladder cancer EVs have also been reported to support the survival of cancer stem-like cells, suggesting that EV-mediated communication may contribute to treatment-tolerant tumour states^[19]^. In addition, bladder cancer EV-associated non-coding RNAs and macrophage-derived EV miR-21 signalling have been linked to epithelial–mesenchymal transition, tumour aggressiveness and immune-related pathways^[20,21]^. More recently, BCG therapy was shown to induce immunogenic EV release from bladder cancer cells; these BCG-induced EVs were enriched in antigen-presentation and co-stimulatory molecules [including major histocompatibility complex (MHC) class I/II, CD80 and CD86] and promoted dendritic-cell activation and T-cell responses^[22]^. These findings support BCG-induced EVs as active mediators of antitumour immune signalling; however, whether altered EV programmes directly contribute to BCG failure or BCG-unresponsive disease remains to be established.

Bladder cancer also provides a relevant setting for translational EV research because urine represents a disease-relevant biofluid for minimally invasive longitudinal monitoring. Urinary EVs can capture molecular signals from tumours and other cells within the urinary tract and are thus attractive candidates for diagnosis, risk stratification and treatment monitoring^[23-28]^. However, urinary EVs are not exclusively tumour-derived, and circulating EVs reflect contributions from tumour, immune, vascular and haematological compartments. These characteristics enable monitoring treatment response and emerging resistance but also complicate source attribution, normalisation and clinical interpretation. This issue is especially relevant in BCG and immunotherapy settings, where EV signals may originate from tumour, immune and stromal cells as well as treatment-induced inflammatory responses.

In this review, we refocus EV-mediated therapeutic resistance on bladder cancer and its major treatments, including BCG-based intravesical immunotherapy, platinum- and gemcitabine-based chemotherapy and immune-checkpoint blockade. We extend the analysis beyond basic descriptions of EV cargo to develop a framework for distinguishing direct EV-mediated drug resistance from treatment-associated EV remodelling, permissive amplification and biomarker associations. We first define the experimental standards required to establish EV-specific causality, including vesicle transfer, cargo-specific perturbation, pathway validation and functional rescue. We then examine EV-associated mechanisms in BCG failure and resistance to chemotherapy and immunotherapy while highlighting preliminary or indirect evidence. Finally, we discuss urinary and circulating EVs as candidates for longitudinal monitoring and outline future directions for drug-specific EV studies in bladder cancer. By integrating therapeutic context, EV causality criteria and bladder cancer biology, this review provides a focused framework for evaluating when EVs mediate drug resistance, amplify adaptive tumour–microenvironment states and primarily serve as biomarkers for therapeutic monitoring.

### Literature identification and selection

Literature for this narrative review was identified through searches of PubMed, Web of Science and Google Scholar up to September 2026. Search terms included combinations of “extracellular vesicles”, “exosomes”, “small extracellular vesicles”, “bladder cancer”, “urothelial carcinoma”, “drug resistance”, “chemotherapy resistance”, “gemcitabine”, “cisplatin”, “platinum-based chemotherapy”, “BCG”, “BCG failure”, “BCG-unresponsive”, “immune-checkpoint blockade”, “PD-L1” and “urinary extracellular vesicles”. Original research articles were prioritised for specific EV-associated mechanisms and mechanistic claims, whereas review articles, clinical guidelines and consensus statements were used mainly for clinical background, conceptual framing and methodological standards. Studies were selected according to their relevance to EV-associated drug resistance, urinary EV biomarkers, bladder cancer treatment contexts and the strength of mechanistic or clinical evidence.

## EVS AS MEDIATORS, AMPLIFIERS AND BIOMARKERS OF BLADDER CANCER DRUG RESISTANCE

EVs provide a mechanistic link between tumour-cell adaptation, microenvironmental communication and treatment responses in bladder cancer. By transferring proteins, nucleic acids, lipids and metabolites, EVs can mediate molecular communication between bladder cancer cells and immune, stromal and endothelial compartments^[13-17]^. In the setting of bladder cancer therapy, this communication should be interpreted in relation to specific treatment pressures, including gemcitabine- and platinum-based chemotherapy, intravesical BCG and immune-checkpoint blockade, rather than as a general feature of therapeutic resistance. This conceptual framework is illustrated in Figure 1.

**Figure 1:**
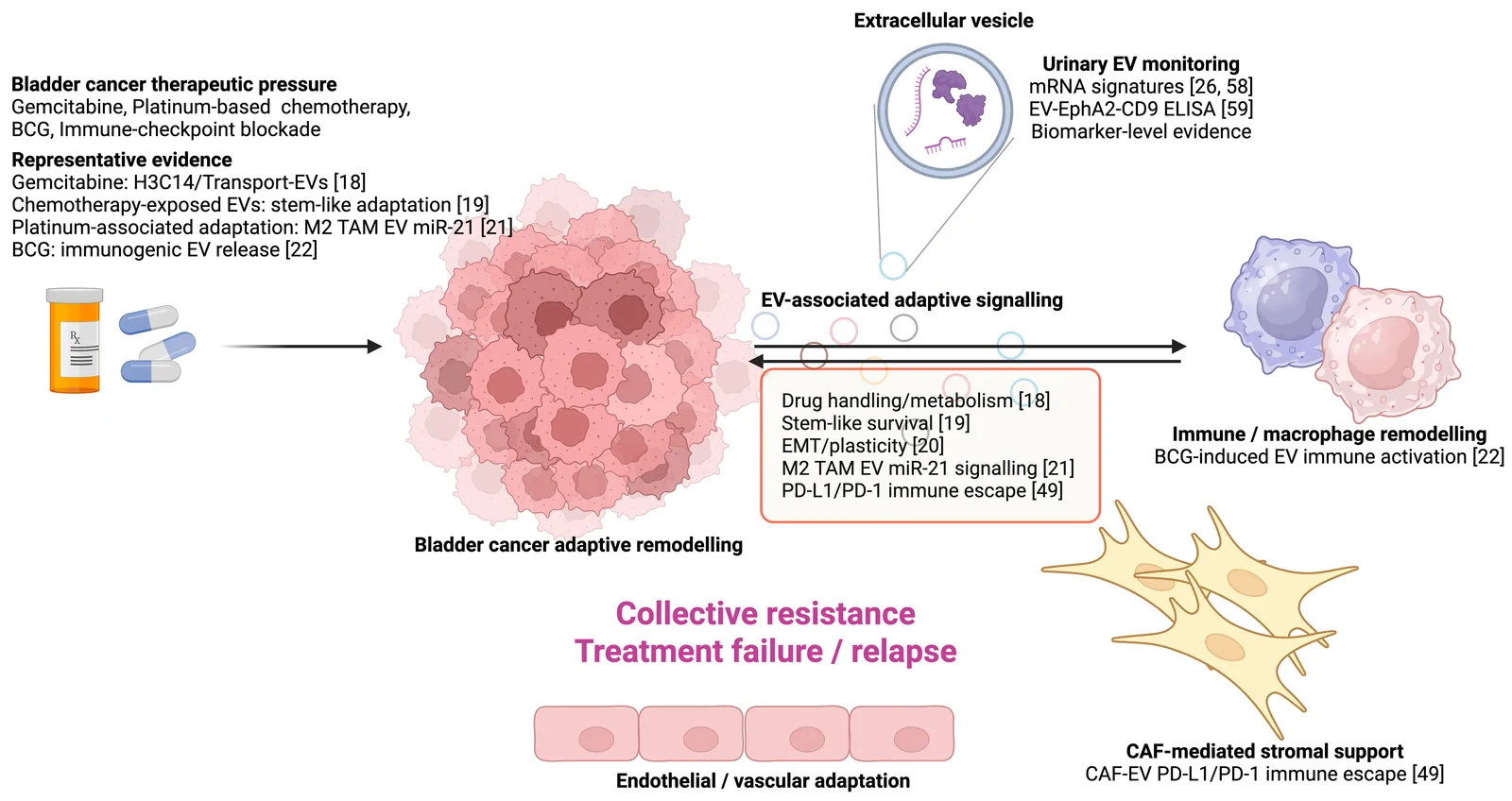
Evidence-mapped framework of EV-associated drug adaptation in bladder cancer. Drug-specific therapeutic pressure, including gemcitabine, platinum-based chemotherapy, BCG therapy and immune-checkpoint blockade, can reshape bladder cancer EV release, cargo composition and tumour–microenvironment communication. Representative bladder cancer studies support EV involvement in gemcitabine-associated H3C14/transport-EV remodelling, chemotherapy-exposed EV-mediated stem-like adaptation, EV-associated non-coding RNA signalling, M2 tumour-associated macrophage-derived EV miR-21 signalling, BCG-induced EV-mediated antitumour immune activation, CAF-derived EV-associated PD-L1/PD-1 signalling and immune escape and urinary EV biomarker monitoring. These mechanisms should be interpreted according to evidence category and strength, distinguishing causal mediators, permissive amplifiers, mechanistic leads and biomarker associations. Created in BioRender. Wu, Y. (2026). https://BioRender.com/lwz0yq8. EV: Extracellular vesicle; BCG: Bacillus Calmette–Guérin; CAF: cancer-associated fibroblast; PD-L1: programmed death-ligand 1; PD-1: programmed cell death protein 1; TAM: tumour-associated macrophage; ELISA: enzyme-linked immunosorbent assay; EMT: epithelial–mesenchymal transition.

An evidence-based interpretation is necessary because EV-associated changes do not necessarily indicate EV-mediated resistance. In some settings, EVs may act as causal mediators by directly transferring cargo that alters drug sensitivity in recipient cells. For example, EV-mediated regulation of H3C14 has been implicated in gemcitabine resistance in bladder cancer, supporting a model in which treatment-adapted EV biology influences chemotherapy sensitivity^[18]^. In other settings, EVs may function as permissive amplifiers, reinforcing adaptive tumour–microenvironment states that support treatment tolerance rather than directly initiating resistance. Chemotherapy-exposed bladder cancer EVs that promote cancer stem-like cell survival provide an example of EV-associated communication that may contribute to treatment-tolerant tumour states^[19]^.

EVs may also serve primarily as biomarkers of treatment-induced adaptation. This distinction is particularly important in bladder cancer because urinary EVs can capture signals from tumour, immune and stromal cells as well as treatment-associated inflammatory responses^[23-28]^. In the context of intravesical BCG therapy, altered EV profiles may reflect immune activation, immune escape, tissue inflammation or tumour adaptation and therefore require functional validation before being interpreted as direct mediators of BCG failure^[22]^.

Within this framework, EVs should be evaluated as context-dependent components of bladder cancer drug adaptation. Depending on their cellular source, vesicle subtype, cargo composition, recipient-cell state and therapeutic timing, EVs may mediate drug resistance, amplify adaptive tumour–microenvironment states or primarily serve as biomarkers for treatment monitoring. This distinction provides the conceptual basis for evaluating EV-associated resistance across gemcitabine- and platinum-based chemotherapy, BCG therapy and immune-checkpoint blockade in bladder cancer.

## EVIDENCE STANDARDS FOR EV-MEDIATED DRUG RESISTANCE IN BLADDER CANCER

Associations between EVs and drug resistance do not by themselves establish an EV-dependent mechanism. In bladder cancer models and biofluids, EVs coexist with soluble cytokines, growth factors, metabolites, ribonucleoprotein complexes, lipoproteins, apoptotic bodies and other extracellular particles. This issue is particularly relevant in the context of drug-specific treatment pressure, given that gemcitabine- and platinum-based chemotherapy, BCG therapy and immune-checkpoint blockade can simultaneously alter tumour cell stress responses, immune signalling, cell death, soluble secretomes and EV release. Therefore, changes in EV abundance, cargo composition or EV uptake should not be interpreted as evidence of EV-mediated drug resistance unless supported by functional and mechanistic validation^[29-31]^.

Overall, three issues should be addressed when evaluating EV-mediated drug resistance in bladder cancer. First, the necessity of EVs for phenotype transmission should be established by disrupting EV biogenesis, release or uptake in donor or recipient cells while controlling for donor-cell viability, non-vesicular secretion and treatment exposure. Second, the sufficiency of well-characterised EVs to reproduce resistance-associated phenotypes in recipient cells should be tested using process-matched controls, including EV-depleted or non-vesicular fractions, dose–response analyses and treatment-specific readouts such as gemcitabine sensitivity, platinum-induced DNA damage, BCG-induced immune activation and immune-checkpoint responses. Third, attribution of these effects to specific EV cargo, pathways or vesicle subtypes requires cargo-specific perturbation, rescue experiments and downstream target validation in recipient cells^[29,30,32-34]^.

Importantly, careful distinctions between EV-mediated effects and parallel signalling processes are necessary. Detection of a candidate molecule within an EV-enriched preparation does not establish that it is membrane-enclosed, functionally delivered or responsible for altered drug responses. Protease- or nuclease-protection assays, density- or size-based fractionation, immunoaffinity enrichment and comparison with EV-depleted or non-vesicular fractions can help determine whether both the candidate cargo and the biological activity depend on membrane-enclosed particles^[29,31]^. Similarly, particle uptake should not be equated with functional delivery of cargo. Stronger evidence can be obtained by demonstrating the regulation of endogenous recipient-cell targets, activation of cargo-dependent reporters and reversal of the phenotype after cargo depletion and rescue^[32,33]^.

The same caution applies to urinary and circulating EV studies. In bladder cancer, urinary EVs are attractive for longitudinal monitoring; however, altered urinary EV cargo may reflect tumour burden, tissue injury, inflammation, treatment-induced immune activation or urinary tract physiology rather than direct resistance signalling^[23-28,35]^. Accordingly, EV signatures in this setting should be interpreted as candidate biomarkers or mechanistic leads unless longitudinal sampling, source attribution and functional validation provide evidence linking specific EV populations to treatment failure.

Ultimately, EV-mediated drug resistance should be inferred from convergent evidence rather than from particle detection, cargo association, uptake imaging or EV add-back experiments alone. Robust evidence requires integrating complementary EV isolation and characterisation methods with drug-specific functional assays, cargo- or subtype-specific perturbation, recipient-cell pathway validation and physiologically relevant models. This evidence hierarchy is essential for distinguishing EVs that actively mediate bladder cancer drug resistance from those that amplify adaptive tumour–microenvironment states or primarily serve as biomarkers of treatment-induced remodelling.

## EV-ASSOCIATED CHEMOTHERAPY RESISTANCE IN BLADDER CANCER

Chemotherapy resistance in bladder cancer involves both stable tumour-cell adaptations and reversible treatment-tolerant states. Gemcitabine- and platinum-based regimens impose selective pressure on tumour cells through effects on DNA synthesis, nucleotide metabolism, DNA damage responses, oxidative stress, apoptosis and survival signalling. In this setting, studies in bladder cancer suggest that EVs may participate in chemotherapy adaptation by redistributing resistance-associated cargo and supporting stem-like tumour-cell survival after chemotherapy exposure^[18,19]^. Nevertheless, the strength of evidence varies across chemotherapy settings, and EV-associated findings should be interpreted in light of drug-specific functional validation. Table 1 summarises representative EV-associated mechanisms and their evidence strength in bladder cancer drug adaptation, along with selected comparative examples from renal cell carcinoma (RCC) and prostate cancer.

**Table 1:** Representative EV-associated mechanisms and evidence strength in bladder cancer drug adaptation, with selected comparative examples from RCC and prostate cancer

**Disease context**	**Therapy/treatment context**	**EV cargo/marker**	**Proposed EV-associated mechanism**	**Evidence category**	**Evidence strength**	**Ref.**
Bladder cancer	Gemcitabine	Transport-EVs marked by CD9, CD63 and/or CD81; excretion-EVs enriched in CD147, LAMB1 and H3.2/H3C14	Transport-EVs propagate resistance-associated changes in nucleoside transporters and metabolic enzymes, whereas CD147/LAMB1-enriched excretion-EVs promote intracellular depletion of the resistance-suppressive histone H3.2/H3C14 from donor cells	Causal mediator	Strong bladder cancer-specific functional evidence	Huang *et al.*, 2025^[18]^
Bladder cancer	Cisplatin and gemcitabine exposure	EV protein cargoes enriched in proteasomal, ribosomal, spliceosomal and ubiquitin-mediated proteolysis pathways; no single causal cargo validated	Chemotherapy-induced NSCC-EVs support proteostasis, stemness and adaptive fitness in recipient CSCs	Permissive amplifier	Functional evidence for chemotherapy-associated EV support of treatment-tolerant stem-like states; cargo-specific causality remains incomplete	Chung *et al.*, 2021^[19]^
Bladder cancer	Not treatment specific	EV-associated LINC00960 and LINC02470	EV-mediated transfer of lncRNAs activates β-catenin, Notch and Smad2/3 signalling, promoting EMT-associated phenotypic plasticity	Mechanistic lead	Functional evidence for EV-associated aggressive phenotypes; no direct evidence of drug resistance	Huang *et al.*, 2020^[20]^
Bladder cancer	Cisplatin-associated adaptation/macrophage-associated EV signalling	M2 TAM-derived EV-associated miR-21 and mRNAs, including those encoding Akt, STAT3, mTOR and β-catenin	Promotes cisplatin resistance, migration and tumour-sphere generation through CDK6, mTOR, STAT3 and β-catenin signalling	Mechanistic lead/permissive amplifier	Functional observation; EV- and cargo-specific causality remains incomplete	Wu *et al.*, 2020^[21]^
RCC comparison	Acquired sunitinib resistance	EV-associated lncARSR, selectively packaged via hnRNPA2B1	EV-transferred lncARSR acts as a competing endogenous RNA for miR-34 and miR-449, increasing AXL and c-MET expression and reactivating STAT3, AKT and ERK signalling	Comparative mechanistic example	Strong functional evidence from RCC model with defined cargo, pathway involvement and therapeutic rescue	Qu *et al.*, 2016^[36]^
RCC comparison	Acquired sunitinib resistance	EV-associated IGFL2-AS1, packaged via hnRNPC in an m6A-dependent manner	EV-transferred IGFL2-AS1 competitively binds hnRNPC, altering TP53INP2 alternative splicing and translation and enhancing TP53INP2-dependent protective autophagy	Comparative mechanistic example	Strong functional and cargo-specific evidence from RCC model	Pan *et al*., 2023^[37]^
RCC comparison	Acquired sorafenib resistance	EV-associated miR-31-5p	Resistant-cell EVs transfer miR-31-5p to sensitive cells, where it suppresses MLH1 and reduces sorafenib responsiveness	Comparative mechanistic example	Functional evidence from an RCC model with defined miRNA cargo	He *et al*., 2020^[38]^
Prostate cancer comparison	First-line abiraterone or enzalutamide in metastatic castration-resistant prostate cancer	EV-associated AR-V7, analysed alongside cfDNA AR gain	EV-associated AR-V7 is associated with persistent ligand-independent AR signalling and may identify tumours with reduced sensitivity to AR signalling inhibitors	Comparative biomarker example	Clinical biomarker association; no evidence of EV-mediated resistance transfer	Del Re *et al.*, 2021^[39]^
Prostate cancer comparison	Acquired enzalutamide resistance	Increased EV output associated with syntaxin 6 upregulation and enhanced syntaxin 6–CD63 colocalisation; no individual resistance-promoting EV cargo validated	Syntaxin 6 promotes EV secretion in enzalutamide-resistant cells, while disruption of syntaxin 6 or EV biogenesis reduces resistant-cell survival during enzalutamide exposure	Comparative mechanistic example	Functional evidence for an EV-secretion-associated resistant state; evidence for horizontal EV transfer and cargo-specific causality remains incomplete	Peak *et al*., 2020^[40]^
Prostate cancer comparison	Taxane chemotherapy resistance, with additional docetaxel- and bicalutamide-resistant models	EV-associated miR-423-5p	CAF-derived EVs transfer miR-423-5p to tumour cells, suppressing GREM2 expression and enhancing TGF-β-associated signalling	Comparative stromal EV example	Functional evidence from prostate cancer model with cargo and pathway-specific support	Shan *et al*., 2020^[41]^

EV: Extracellular vesicle; RCC: renal cell carcinoma; NSCC: non-stem cancer cell; CSCs: cancer stem cells; EMT: epithelial–mesenchymal transition; TAM: tumour-associated macrophage; Akt: protein kinase B; STAT3: signal transducer and activator of transcription 3; mTOR: mechanistic target of rapamycin; AXL: AXL receptor tyrosine kinase; c-MET: cellular mesenchymal–epithelial transition factor; ERK: extracellular signal-regulated kinase; MLH1: MutL homolog 1; AR: androgen receptor; CAF: cancer-associated fibroblast; GREM2: Gremlin 2; TGF-β: transforming growth factor beta.

### Gemcitabine resistance and EV-associated adaptive remodelling

Among bladder cancer chemotherapy models, gemcitabine resistance currently provides a well-characterised setting in which EV-associated mechanisms have been functionally examined. EV-mediated regulation of H3C14 has been implicated in gemcitabine resistance in bladder cancer, suggesting that altered EV biology can influence drug sensitivity and resistance-associated cellular phenotypes^[18]^. This finding supports a model in which gemcitabine-resistant bladder cancer cells undergo not only intracellular adaptation but also remodelling of EV-associated cargo and EV-mediated communication.

The biological interpretation of these EV-associated changes warrants caution. EV-associated H3C14 or related cargo should not be viewed simply as isolated resistance factors; rather, they may reflect broader treatment-associated remodelling involving nucleotide metabolism, chromatin-associated proteins, vesicle subtype composition and recipient-cell responses. More broadly, EV studies support the concept that EVs can transfer solute transporters and metabolic regulators that alter bioenergetic dependence and treatment response, but these mechanisms require direct validation in bladder cancer chemotherapy models^[42]^. Therefore, gemcitabine-associated EV mechanisms should be evaluated through cargo-specific perturbation, comparison of EV subpopulations and functional rescue experiments. In this setting, EVs may represent functionally relevant mediators of gemcitabine resistance when EV depletion or cargo-specific manipulation alters drug response, whereas EV cargo alterations without functional validation should be interpreted as treatment-associated remodelling or candidate biomarkers.

### Chemotherapy-exposed EVs and treatment-tolerant tumour states

Chemotherapy can generate EV populations that promote treatment-tolerant or stem-like tumour states. In bladder cancer, non-stem cancer cell-derived EVs have been reported to promote cancer stem cell survival in response to chemotherapy, indicating that EV-mediated communication may help maintain a cellular reservoir capable of surviving cytotoxic stress^[19]^. Importantly, this observation links chemotherapy exposure to EV-mediated maintenance of a stem-like compartment, providing a potential mechanism through which EVs may contribute to non-genetic treatment tolerance in bladder cancer.

This model is particularly relevant for recurrent bladder cancer, where residual tumour cells may persist after initial treatment and subsequently contribute to disease relapse. EV-associated support of cancer stem-like or drug-tolerant states may involve altered survival signalling, metabolic adaptation, stress-response pathways, non-coding RNA transfer and modulation of local tumour–microenvironment communication. Nevertheless, whether chemotherapy-exposed EVs initiate resistance, maintain residual disease or simply accompany chemotherapy-induced stress remains unclear. Future studies should integrate time-course sampling, chemotherapy rechallenge, EV depletion and rescue experiments to elucidate whether chemotherapy-exposed EVs are necessary and sufficient to sustain treatment-tolerant bladder cancer populations.

### Platinum-based chemotherapy, DNA damage tolerance and cell-death evasion

Compared with gemcitabine-associated EV studies, direct evidence for EV-mediated platinum resistance in bladder cancer remains limited. Platinum-based chemotherapy primarily induces DNA damage and cytotoxic stress, and resistant bladder cancer cells may adapt through enhanced DNA repair, altered apoptosis, redox regulation, drug transport and phenotypic plasticity. Although evidence from the broader EV literature implicates EV-associated cargo in DNA damage responses, oxidative stress, autophagy and apoptosis, direct evidence that EVs mediate platinum resistance in bladder cancer is not yet well established^[43-45]^.

Nevertheless, available bladder cancer studies provide functional observations relevant to platinum-associated adaptation. For example, macrophage-derived EV-enriched preparations carrying miR-21-5p and related RNA cargoes have been linked to activation of protein kinase B (Akt), signal transducer and activator of transcription 3 (STAT3), mechanistic target of rapamycin (mTOR), β-catenin, cyclin-dependent kinase 6 (CDK6) and interleukin-6 (IL-6) signalling, together with stem-like adaptation and reduced cisplatin sensitivity in bladder cancer cells^[21]^. These findings support a plausible tumour–microenvironment EV-associated mechanism that may contribute to cisplatin tolerance. However, EV-specific causality, vesicle subtype resolution and cargo-specific attribution remain incompletely established, and the contribution of individual EV cargoes to platinum response requires further perturbation and rescue experiments.

For platinum resistance, informative experimental designs should determine whether EVs from platinum-exposed or platinum-resistant bladder cancer cells alter cisplatin sensitivity, DNA damage markers, apoptosis, clonogenic survival or recovery following drug withdrawal in recipient cells. Stronger causal inference would require demonstrating that inhibition of EV pathways reduces resistance transfer, that isolated EVs reproduce the phenotype under physiologically relevant conditions, and that cargo-specific perturbation or rescue modifies platinum response. In the absence of such evidence, EVs in platinum-treated bladder cancer should be described as candidate modulators or mechanistic leads in chemotherapy adaptation rather than established mediators of platinum resistance.

### Non-coding RNAs, epithelial–mesenchymal transition and chemotherapy-associated phenotypic plasticity

EV-associated non-coding RNAs may contribute to phenotypic plasticity in bladder cancer, although their direct roles in chemotherapy resistance remain incompletely defined. Bladder cancer cell-derived EV-associated LINC00960 and LINC02470 have been linked to epithelial–mesenchymal transition and aggressive phenotypes^[20]^. In addition, macrophage-derived EV miR-21 signalling has been implicated in bladder carcinogenesis through pathways involving mTOR, β-catenin and CDK6^[21]^. These findings support the broader concept that EV-associated RNA signalling can influence bladder cancer cell state, tumour aggressiveness and tumour–microenvironment communication.

Intracellular miRNA pathways also provide important mechanistic context for miRNA-mediated regulation of bladder cancer biology. For example, the miR-143/MSI2/KRAS cascade has been implicated in regulating bladder cancer cell growth^[46]^. However, such intracellular miRNA mechanisms should be distinguished from EV-mediated RNA transfer. Their relevance to EV-mediated drug resistance would require evidence of EV-dependent packaging, vesicular delivery to recipient cells and effects on treatment sensitivity. Without such evidence, these intracellular miRNA pathways should be considered background biology rather than evidence of EV-mediated drug resistance.

This distinction is crucial because epithelial–mesenchymal transition (EMT), stem-like properties and aggressive growth are often associated with reduced treatment sensitivity, although these associations do not necessarily indicate EV-mediated drug resistance. To establish a chemotherapy-specific mechanism, future studies should determine whether EV-associated non-coding RNAs directly alter gemcitabine or platinum response, whether their depletion from donor cells reduces resistance transfer and whether restoration of downstream targets reverses the resistant phenotype. In the absence of such evidence, these EV-associated RNA programmes should be interpreted as mechanistic leads linking EV communication to phenotypic plasticity rather than as definitive drivers of chemotherapy resistance.

### Summary

Taken together, current evidence suggests that EVs may contribute to chemotherapy adaptation in bladder cancer through several non-mutually exclusive mechanisms: redistribution of resistance-associated cargo; support of stem-like or treatment-tolerant states; modulation of DNA damage tolerance and cell-death responses; and reinforcement of phenotypic plasticity. Bladder cancer-specific evidence is currently most developed for gemcitabine-associated EV remodelling and chemotherapy-exposed EV support of cancer stem-like survival^[18,19]^. Evidence for platinum-specific EV-mediated resistance remains comparatively indirect and requires dedicated functional validation. Therefore, EVs should be viewed as context-dependent contributors to chemotherapy adaptation in bladder cancer rather than as uniformly causal drivers of drug resistance.

## EVS IN BCG FAILURE AND IMMUNOTHERAPY RESISTANCE

BCG-based intravesical immunotherapy and immune-checkpoint blockade represent immune-centred therapeutic contexts in bladder cancer. Unlike chemotherapy resistance, which is often examined through tumour-cell survival after drug exposure, resistance to immunotherapy depends on dynamic interactions among tumour cells, antigen-presenting cells, lymphocytes, suppressive myeloid cells, stromal cells and soluble inflammatory mediators^[1,3]^. EVs may participate in these interactions by transferring antigen-presentation molecules, immune-regulatory proteins, non-coding RNAs and inflammatory cargo between tumour and immune compartments^[12,15,22]^. However, EV-associated immune changes in bladder cancer should be interpreted cautiously because they may reflect antitumour immune activation, immune escape, treatment-induced inflammation or tissue injury rather than direct evidence of EV-mediated resistance.

### BCG-induced EVs and antitumour immune signalling

Although BCG remains a central intravesical therapy for intermediate- and high-risk NMIBC, treatment failure or relapse occurs in a substantial proportion of patients. BCG-unresponsive disease is clinically important because further BCG therapy is unlikely to provide meaningful benefit, and bladder-sparing alternatives are increasingly being evaluated for patients who are unfit for or decline radical cystectomy^[2,3,12]^. These clinical features make BCG-treated NMIBC a relevant setting for investigating EV-associated tumour–immune communication.

Recent evidence directly supports a role for BCG-induced EVs in antitumour immune signalling. In bladder cancer models, BCG internalisation by bladder cancer cells was associated with increased EV secretion and changes in EV cargo composition. These BCG-induced EVs were enriched in immunomodulatory molecules, including MHC class I and II molecules and the co-stimulatory molecules CD80 and CD86, and were shown to activate dendritic cells and enhance T-cell responses^[22]^. *In vivo*, administration of BCG-induced EVs enhanced the antitumour efficacy of BCG in a syngeneic murine bladder cancer model^[22]^. These findings support a potential role for EVs as active intermediaries of BCG-induced antitumour immunity rather than simply as byproducts of BCG exposure.

Differences in EV profiles between BCG-responsive and BCG-unresponsive tumours, their temporal association with clinical failure and their functional contribution to BCG response remain to be established. Defining these relationships will help determine whether EVs function primarily as biomarkers, permissive amplifiers or causal mediators in BCG-treated NMIBC.

### BCG-derived bacterial EVs and vesicular immunotherapy concepts

In addition to tumour-cell-derived EVs induced by BCG exposure, bacterial EVs derived from *Mycobacterium bovis* BCG represent a related but mechanistically distinct vesicular system. BCG-derived EVs have been investigated as potential alternatives to live BCG immunotherapy^[47]^. These vesicles may retain selected immunostimulatory properties of BCG while potentially avoiding some limitations associated with live bacterial administration. These findings support the broader concept that vesicular products can participate in BCG-associated immune activation.

BCG-derived bacterial EVs should be distinguished from bladder cancer cell-derived EVs and host immune-cell-derived EVs, given their distinct cellular origin, cargo composition, biodistribution and mechanisms of immune activation. In this review, we discuss these vesicles as a complementary approach to vesicular immunotherapy rather than as direct evidence of host EV-mediated resistance in bladder cancer.

### BCG-unresponsive disease and EV-associated immune escape

BCG-unresponsive NMIBC reflects a complex immune-evasive state within the bladder tumour-microenvironment. Proposed mechanisms include suppressive myeloid cells, tumour-associated macrophages, regulatory T cells, tolerogenic dendritic cells, programmed cell death protein 1 (PD-1)/programmed death-ligand 1 (PD-L1) signalling, NKG2A/HLA-E signalling, effector T-cell exhaustion and CAFs^[48]^. These mechanisms can impair antigen presentation, suppress cytotoxic immune responses and limit sustained antitumour immunity, thereby undermining the efficacy of BCG therapy^[3]^.

EVs may represent one component of this immune-evasion network, particularly through stromal and immune-regulatory communication. In bladder cancer, CAF-derived EVs have been reported to promote immune escape through PD-L1/PD-1-associated signalling, with increased PD-L1 expression, reduced CD8^+^ T-cell infiltration and enhanced tumour growth in experimental models^[49]^. These findings provide bladder cancer-specific evidence linking EV-mediated stromal communication to immune suppression. However, they should be interpreted as evidence of EV-associated immune escape rather than direct evidence of EV-mediated BCG failure, as these studies were not specifically designed to model BCG-unresponsive NMIBC.

Rigorous interpretation of EVs in BCG-unresponsive disease warrants longitudinal, source-resolved studies. Urinary or tissue-associated EV populations should be profiled before BCG, during induction and maintenance therapy, and at recurrence or treatment failure. Such studies should determine whether EV cargo changes originate from tumour cells, immune cells, stromal cells or BCG-associated inflammatory responses, and whether specific EV populations functionally influence dendritic-cell activation, T-cell exhaustion, macrophage polarisation or PD-1/PD-L1 signalling. Pending such evidence, EV signatures in BCG-unresponsive NMIBC should be considered candidate biomarkers or mechanistic leads rather than established drivers of BCG resistance.

### EVs and immune-checkpoint blockade

Immune-checkpoint blockade has become an important component of systemic therapy for advanced urothelial carcinoma and is also used in selected BCG-unresponsive NMIBC settings^[5]^. Because responses to checkpoint blockade depend on tumour antigenicity, immune-cell activation, suppressive myeloid populations, T-cell exhaustion and PD-1/PD-L1 signalling, EVs are biologically plausible candidates as modulators or biomarkers of treatment response^[12,50]^. EV-associated immune regulators, including EV-bound checkpoint-related proteins, may reflect the balance between antitumour immunity and immune suppression.

Among EV-associated immune-checkpoint molecules, PD-L1 is particularly relevant to bladder cancer immunotherapy. Studies in bladder and urothelial cancer have reported EV-associated PD-L1 in both experimental and clinical contexts, including stromal EV-mediated modulation of PD-1/PD-L1 signalling and urinary EV-associated PD-L1 as a candidate biomarker^[49]^. These findings support the biological relevance and potential translational utility of EV-associated PD-L1 in bladder and urothelial cancer. Nevertheless, current evidence remains insufficient to define EV-associated PD-L1 as an established mediator of resistance to immune-checkpoint blockade^[51]^. In particular, EV-bound PD-L1 should be distinguished from soluble PD-L1 and tissue PD-L1 expression, and the cellular origin of EV-associated PD-L1, including tumour, stromal and immune compartments, requires further clarification.

Evidence from broader solid-tumour studies further suggests that soluble and EV-bound immune molecules may have distinct implications as biomarkers during immunotherapy^[50]^. In bladder cancer, such observations support the rationale for analysing EV-associated immune cargo, but should not be interpreted as evidence of EV-mediated resistance to immune-checkpoint blockade. Future bladder cancer studies should evaluate whether EV-associated PD-L1, CD27 or other immune-regulatory molecules predict response to immune-checkpoint blockade independently of tumour burden, soluble biomarkers, tissue PD-L1 expression and immune-cell infiltration.

### Summary

Current evidence supports a context-dependent role for EVs in bladder cancer immunotherapy. BCG-induced tumour-cell EVs can promote antitumour immune signalling and may contribute to BCG therapeutic efficacy^[22]^. BCG-derived bacterial EVs represent a complementary vesicular immunotherapy approach^[47]^. In contrast, direct evidence that endogenous EVs mediate BCG failure or resistance to immune-checkpoint blockade remains incompletely established. BCG-unresponsive disease is shaped by a complex immune-evasion network, and EVs may act as candidate contributors, amplifiers or biomarkers within this network rather than as isolated causal drivers^[3,7]^. Future studies should integrate longitudinal urinary EV profiling, tissue-based EV source attribution and functional immune assays to determine whether specific EV populations actively influence BCG response or sensitivity to immune-checkpoint blockade in bladder cancer.

## COMPARATIVE INSIGHTS FROM RCC AND PROSTATE CANCER

Although this review specifically focuses on bladder cancer, selected observations from RCC and prostate cancer can provide useful comparative insights into the interpretation of EV-associated drug resistance. These malignancies differ substantially in dominant therapeutic pressures, tumour lineage biology and microenvironmental architecture, including hypoxia- and angiogenesis-driven adaptation in RCC and androgen receptor (AR)-dependent lineage plasticity in prostate cancer^[52-56]^. Therefore, evidence from RCC or prostate cancer should not be directly extrapolated to bladder cancer. Nevertheless, these studies illustrate experimental strategies and mechanistic standards that may help guide future EV research in bladder cancer. These disease-specific EV landscapes are illustrated in Figure 2, with bladder cancer emphasised as the central focus and RCC and prostate cancer presented as comparative references.

**Figure 2:**
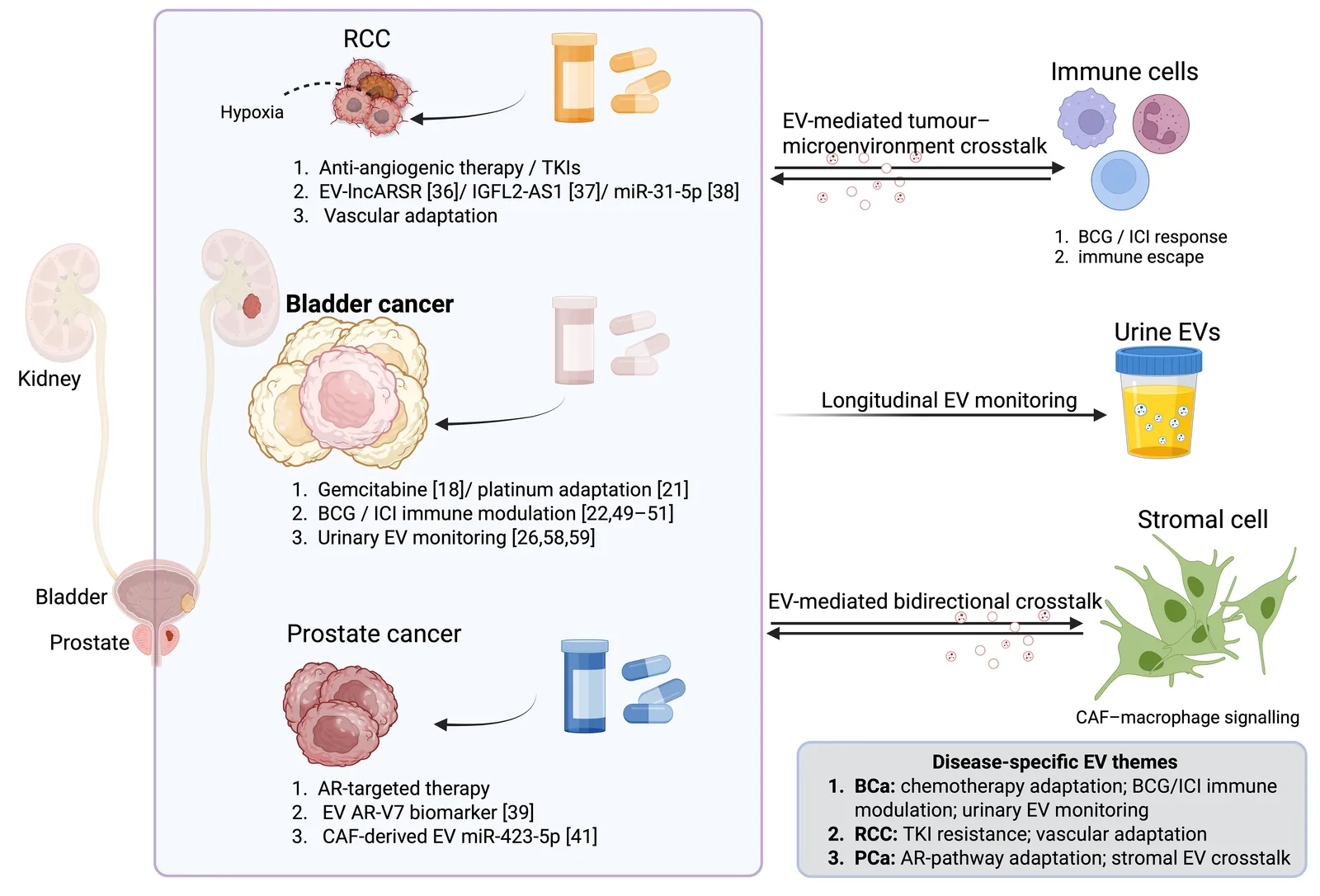
Comparative EV-associated resistance themes across bladder cancer, RCC and prostate cancer. Although this review focuses on bladder cancer, selected studies in RCC and prostate cancer provide comparative insights into the role of EVs in treatment adaptation. In RCC, representative evidence supports EV-associated TKI resistance mediated by lncARSR, IGFL2-AS1 and miR-31-5p, as well as hypoxia-associated vascular adaptation. In bladder cancer, EV studies support chemotherapy-associated adaptation, BCG- and immune-checkpoint blockade-associated immune modulation and the emerging role of urinary EV monitoring. In prostate cancer, EV-associated themes include AR-pathway adaptation, EV-associated AR-V7 as a biomarker of resistance to AR-targeted therapy and stromal EV-mediated crosstalk. The figure summarises representative disease-specific EV themes rather than implying equivalent levels of evidence across tumour types. Created in BioRender. Wu, Y. (2026) https://BioRender.com/rrmpdf1. EV: Extracellular vesicle; RCC: renal cell carcinoma; TKI: tyrosine kinase inhibitor; BCG: Bacillus Calmette–Guérin; AR: androgen receptor; ICI: immune-checkpoint inhibitor; CAF: cancer-associated fibroblast.

RCC currently provides well-characterised mechanistic examples of EV-mediated therapeutic resistance among urological malignancies. In sunitinib-resistant RCC models, EV-associated lncARSR was transferred from resistant to sensitive cells and promoted resistance through a miR-34/miR-449–AXL/c-MET signalling axis^[36]^. In addition, EV-associated IGFL2-AS1 was reported to promote sunitinib resistance by regulating TP53INP2-related alternative splicing and protective autophagy^[37]^. EV-transmitted miR-31-5p has also been implicated in sorafenib resistance through MLH1 suppression^[38]^. These studies are particularly relevant because they combine resistant-donor and sensitive-recipient models, cargo-specific perturbation, downstream pathway validation and, in some cases, *in vivo* or clinical evidence. Compared with the current bladder cancer evidence base, RCC studies highlight how stronger causal inference can be achieved when EV transfer is linked to a defined drug, cargo and rescue pathway.

Prostate cancer provides a different comparison, centred on AR signalling, AR-targeted therapy and stromal cooperation. EVs released under androgen-stimulated or androgen-deprived conditions can alter recipient-cell growth or reflect therapy-associated remodelling^[40,57]^. Circulating EV-enriched AR-V7 RNA has been associated with poorer outcomes in patients receiving abiraterone or enzalutamide, supporting the potential utility of EVs as biomarkers of persistent AR-pathway activity rather than providing direct evidence of EV-mediated resistance transfer^[39]^. Further mechanistic evidence is provided by CAF-derived EVs carrying miR-423-5p, which reduced drug sensitivity through Gremlin 2 (GREM2) suppression and transforming growth factor beta (TGF-β)-associated signalling; partial reversal by donor-cell miR-423-5p inhibition, GREM2 restoration and TGF-β blockade strengthened cargo- and pathway-specific inference^[41]^.

These comparative examples reinforce three principles relevant to bladder cancer. First, EV-mediated resistance should be interpreted within a defined therapeutic context, such as gemcitabine, platinum-based chemotherapy, BCG or immune-checkpoint blockade. Second, biomarker associations, treatment-induced EV remodelling and EV-dependent resistance transfer should be analytically distinguished. Third, robust causal evidence requires defined EV source, cargo-specific perturbation, recipient-cell pathway validation and functional rescue. In bladder cancer, such standards will be particularly important for distinguishing EVs that actively mediate chemotherapy resistance or immunotherapy failure from EVs that primarily reflect treatment-induced tumour evolution, inflammation or urinary tract injury.

## LONGITUDINAL EV MONITORING OF BLADDER CANCER DRUG ADAPTATION

The accessibility of EVs in urine and blood enables repeated, minimally invasive assessment of treatment-associated molecular changes in bladder cancer^[23-28]^. Unlike tissue biopsy, which provides a spatially and temporally restricted snapshot, longitudinal EV profiling may capture dynamic changes in tumour, immune and stromal states during therapy. This approach is particularly relevant for bladder cancer because urinary EVs can be sampled serially during intravesical BCG, chemotherapy, surveillance and systemic therapies. However, the clinical relevance of an EV signal depends on the treatment context, timing of sampling and intended clinical application. As illustrated in Figure 3, EV-based assessment can be conceptualised across three complementary windows: pretreatment stratification, early on-treatment monitoring and detection of molecular changes preceding overt clinical resistance. Each application requires distinct study designs, analytical thresholds and validation criteria and should therefore not be considered interchangeable.

**Figure 3:**
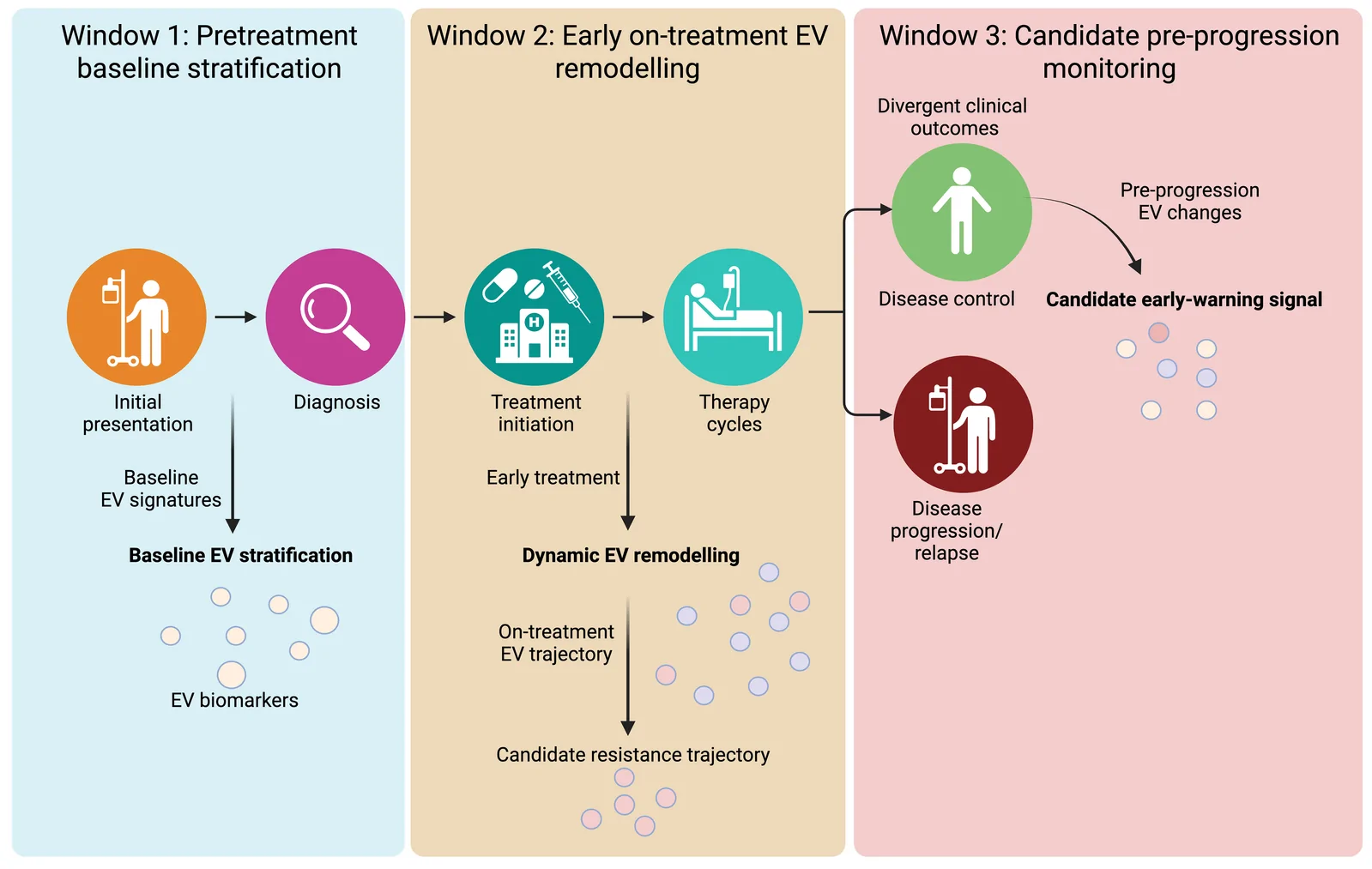
Longitudinal EV monitoring windows in bladder cancer drug adaptation. EV-based assessment in bladder cancer can be conceptualised across three complementary clinical windows: pretreatment baseline stratification, early on-treatment EV remodelling and candidate pre-progression monitoring. Pretreatment EV profiles may support baseline risk stratification but should not be considered validated treatment-selection tools without treatment-specific validation. Early on-treatment EV trajectories may reflect pharmacodynamic response, treatment-induced injury, immune activation or emerging adaptive states. Candidate pre-progression EV changes may provide early-warning signals before overt clinical resistance, but their clinical use warrants prespecified signatures, serial sampling, source attribution and independent validation. These windows should be interpreted according to treatment context, including gemcitabine, platinum-based chemotherapy, BCG and immune-checkpoint blockade. Created in BioRender. Wu, Y. (2026) https://BioRender.com/25batum. EV: Extracellular vesicle; BCG: Bacillus Calmette–Guérin.

### Representative urinary EV biomarker studies in bladder cancer

Representative urinary EV biomarker studies illustrate both the promise and current limitations of EV-based monitoring in bladder cancer. For example, Sun *et al.* identified a urinary EV mRNA signature for bladder cancer using EV transcriptomic sequencing from normal and bladder cancer cell lines, followed by reverse transcription-quantitative polymerase chain reaction (RT-qPCR) validation in clinical urine samples. The three-mRNA panel, consisting of SRGN, FLI1 and MACROH2A2, showed diagnostic performance and potential prognostic relevance, and demonstrated reduced postoperative signal, supporting the feasibility of urinary EV mRNA profiling as a non-invasive biomarker strategy^[26]^. However, this study was primarily diagnostic and perioperative rather than designed around serial monitoring during BCG therapy, chemotherapy or immune-checkpoint blockade.

In a more longitudinally oriented study, Murakami *et al.* evaluated urinary EV mRNA markers, including KRT17, GPRC5A, SLC2A1, MDK and CXCR2, using RT-qPCR analysis with ALDOB normalisation in cross-sectional and longitudinal urothelial bladder cancer cohorts. The authors found that urinary EV mRNA markers could support bladder cancer detection and that changes in KRT17 levels were associated with recurrence status during follow-up. These findings are directly relevant to longitudinal EV monitoring, although prospective validation in treatment-specific settings such as BCG failure or chemotherapy adaptation is still needed^[58]^.

Urinary EV protein-based assays further illustrate the translational potential of clinically adaptable EV platforms. Tomiyama *et al.* identified EphA2 as a urinary EV protein biomarker and developed an EV-EphA2-CD9 enzyme-linked immunosorbent assay (ELISA) for urinary EV-EphA2 measurement without ultracentrifugation or mass spectrometry. Although these findings support the feasibility of EV protein assay development for bladder cancer detection, the study remains primarily diagnostic and has yet to establish whether urinary EV-EphA2 dynamics reflect treatment response, recurrence risk during surveillance or drug-resistant adaptation^[59]^.

### Window 1: Baseline predictive stratification

Pretreatment EV profiles may identify molecular features associated with baseline treatment sensitivity, pre-existing resistant states or risk of subsequent therapeutic failure. In bladder cancer, candidate EV analytes include tumour-associated RNAs, proteins, glycoproteins, surface markers and immune-regulatory molecules detected in urinary or circulating EVs^[23-28]^. For chemotherapy, baseline EV profiles may eventually help identify tumours with pre-existing gemcitabine or platinum tolerance. For BCG or immune-checkpoint blockade, pretreatment EV signatures may reflect tumour immunogenicity, immune suppression or baseline inflammatory states.

However, an association with outcome does not establish predictive utility. A prognostic EV marker reflects the expected clinical course irrespective of treatment, whereas a predictive marker identifies differential benefit from a specific therapy. Accordingly, pretreatment EV studies should be conducted within well-defined bladder cancer treatment cohorts, such as gemcitabine/platinum-treated MIBC, BCG-treated NMIBC or immune-checkpoint inhibitor-treated advanced disease. Where possible, they should incorporate comparator groups and determine whether EV-derived biomarkers provide incremental value beyond tumour burden, disease stage, histopathological features, urine cytology, imaging and established molecular variables. Within the framework shown in Figure 3, Window 1 represents a candidate approach to baseline stratification rather than an established EV-guided treatment-selection strategy.

### Window 2: Early on-treatment EV remodelling

Serial EV sampling during early treatment may reveal molecular changes that are not captured by pretreatment profiling. In bladder cancer, this window is especially relevant during BCG induction, chemotherapy cycles and early immune-checkpoint blockade. EV abundance, cargo composition and cellular origin may shift in response to drug-induced tumour stress, immune activation, tissue injury, inflammatory remodelling or the emergence of treatment-tolerant cellular states. Such changes should not be interpreted automatically as evidence of therapeutic resistance.

The clinical value of this window depends on whether serial EV trajectories can distinguish favourable treatment response from adaptive changes that precede therapeutic failure. For chemotherapy, relevant readouts may include EV-associated markers of gemcitabine adaptation, DNA damage tolerance, apoptosis resistance or stem-like tumour-cell survival^[18,19]^. For BCG-treated NMIBC, early EV remodelling may reflect antitumour immune activation, as suggested by BCG-induced EVs enriched in MHC class I/II, CD80 and CD86, but may also reflect treatment-associated inflammation or emerging immune escape^[3,22]^. For immune-checkpoint blockade, EV-bound immune molecules may provide complementary information to soluble biomarkers, but their utility requires treatment-specific validation^[50]^.

Preanalytical variation remains a major limitation, particularly for urinary EV analysis. Collection conditions, stabilisation, storage, concentration and normalisation can substantially influence measured EV abundance and cargo profiles^[35]^. Apparent on-treatment changes may also arise from urinary dilution, renal dysfunction, haematuria, infection, inflammation or tissue injury rather than tumour adaptation. Accordingly, longitudinal studies should incorporate standardised urine and blood collection protocols, appropriate normalisation strategies, source-attribution methods and relevant clinical covariates. Within the framework shown in Figure 3, Window 2 represents a candidate pharmacodynamic and adaptive-monitoring phase rather than an established basis for treatment modification.

### Window 3: Candidate molecular progression before overt clinical resistance

A major potential application of longitudinal EV analysis is the detection of molecular changes that precede radiographic, cystoscopic or pathological progression. In bladder cancer, this may include EV changes that emerge before recurrence after BCG, progression from NMIBC to MIBC, relapse after perioperative chemotherapy or progression during systemic immunotherapy. As illustrated in Figure 3, this window represents a candidate pre-progression phase in which evolving EV profiles may provide an early indication of treatment escape.

Nonetheless, current evidence does not establish whether EV alterations consistently precede overt resistance in bladder cancer. Demonstrating an early-warning role requires serial sampling, a prespecified EV signature, evidence that the EV signal emerges before progression and validation in an independent treatment-specific cohort. The candidate EV signal should also provide incremental information beyond conventional imaging, cystoscopy, urine cytology, circulating tumour DNA, established serum or urinary biomarkers and relevant clinical variables. EV-associated and soluble forms of the same molecule should be analysed separately because they may convey distinct biological and clinical information; for example, soluble and EV-bound CD27 have shown opposing associations in patients receiving immunotherapy in a broader solid-tumour context^[50]^.

Once these criteria have been satisfied, a pre-progression EV change should be viewed as a potential basis for treatment intensification, combination therapy or mechanism-informed therapeutic modification. Pending such validation, EV changes observed before clinical progression should be regarded as candidate molecular warning signals rather than validated triggers for clinical intervention. Within the roadmap shown in Figure 3, Window 3 represents a proposed interception opportunity rather than an established EV-guided treatment strategy.

## TECHNICAL AND TRANSLATIONAL CHALLENGES IN BLADDER CANCER EV RESEARCH

The reproducibility and clinical translation of EV research in bladder cancer remain constrained by preanalytical, analytical and biological heterogeneity. Differences in sample collection, EV enrichment, characterisation, normalisation, experimental dosing and outcome assessment can substantially alter molecular profiles and functional conclusions^[29,30]^. These limitations are especially relevant to drug-resistance studies because treatment itself can alter cell viability, EV release, cargo composition and the relative abundance of vesicular and non-vesicular extracellular components. In bladder cancer, additional complexity arises from the use of urine as a major biofluid source, repeated intravesical therapy, haematuria, infection, inflammation and variable renal or urinary tract conditions.

### Biofluid variability and EV source attribution

Blood- and urine-derived EV measurements are highly sensitive to collection and processing conditions. In blood, platelet activation, haemolysis, coagulation, processing delay and residual lipoproteins can alter EV abundance and molecular profiles. Urinary EV analysis is additionally influenced by hydration, urine concentration, renal function, inflammation, collection timing, uromodulin, storage conditions and freeze–thaw cycles^[25,35]^. These variables are particularly important in bladder cancer, where urine may contain EVs and other particles derived from tumour cells, non-malignant urothelium, renal and urinary tract epithelia, inflammatory cells, immune infiltrates and treatment-associated tissue injury.

These variables can obscure biologically meaningful changes, particularly in serial studies during BCG, chemotherapy or immunotherapy. Treatment may induce apoptosis, inflammation, vascular injury, platelet activation or organ dysfunction, thereby altering EV release and increasing the contribution of non-tumour-derived EVs and other extracellular particles. Consequently, resistance-associated EV signatures require standardised processing and, where possible, source attribution using tumour-associated molecular alterations, surface markers, cell-type-selective enrichment or paired tissue-based analyses. Interpretation should also account for tumour burden, treatment timing, organ function, urinary tract infection, haematuria and concurrent inflammatory conditions.

### Isolation, heterogeneity and normalisation

No single isolation method recovers all EV populations with equivalent purity and yield. Ultracentrifugation, size-exclusion chromatography, precipitation, density-based separation and immunoaffinity capture enrich overlapping but non-identical particle populations and differ in susceptibility to contamination by soluble proteins, lipoproteins, ribonucleoproteins and membrane fragments^[29-31]^. Therefore, method-dependent differences can influence both cargo profiling and functional assays.

EV heterogeneity further complicates interpretation. Tumour, immune, stromal and endothelial cells release vesicle populations that differ in biogenesis, surface composition, cargo and biological activity. Bulk EV profiling may dilute rare resistance-associated subsets or incorrectly attribute their activity to the broader EV population. This issue is especially relevant for bladder cancer drug adaptation, where functionally distinct EV populations may have divergent roles in chemotherapy resistance, BCG-induced immune activation or immune escape. Subtype-selective enrichment, multiplexed surface profiling and single-vesicle analysis may improve resolution, but these approaches remain technically demanding and incompletely standardised.

Accordingly, EV quantification should incorporate complementary normalisation metrics whenever feasible. Particle number, total protein, input biofluid volume, donor-cell number and donor-cell equivalents each capture different aspects of EV abundance, but no single metric defines purity, cellular origin or functional competence^[34]^. Functional studies should report EV dose, exposure duration, donor-cell viability, recipient-cell number and the relationship between experimental EV dosing and physiologically plausible exposure levels.

### Experimental models and drug-specific context

Most mechanistic studies rely on two-dimensional culture systems, which do not fully recapitulate the cellular diversity, extracellular matrix, vascular architecture or pharmacological environment of human bladder tumours. EV production, distribution and uptake may differ in three-dimensional cultures, organoids, animal models and patient-derived systems. Patient-derived organoids and xenografts can more closely recapitulate molecular, histopathological and drug-response features of the original bladder tumours, making them useful complementary platforms for therapy-oriented mechanistic studies^[60,61]^. Likewise, acute exposure to high drug concentrations may not mimic prolonged or intermittent clinical treatment schedules, including intravesical BCG induction and maintenance, repeated chemotherapy cycles or immune-checkpoint blockade.

Therefore, experimental design should reflect the relevant drug class, dose, exposure duration, treatment cycle and disease stage. For gemcitabine and platinum-based chemotherapy, models should evaluate drug sensitivity, DNA damage, apoptosis, clonogenic recovery and treatment-tolerant cell states. For BCG and immune-checkpoint blockade, models should incorporate tumour–immune interactions, antigen presentation, dendritic-cell activation, T-cell exhaustion, macrophage polarisation and PD-1/PD-L1 signalling. Findings from exogenous EV add-back experiments also warrant cautious interpretation, as administered vesicles may not reproduce endogenous concentrations, biodistribution, clearance or recipient-cell tropism.

### Clinical validation and reproducibility

Many proposed EV biomarkers are derived from small, retrospective or single-centre cohorts, with limited external replication. Differences in sample handling, isolation platforms, analytical thresholds and clinical endpoints further hinder comparability. Biomarker performance is also frequently reported without demonstrating incremental value beyond conventional clinical and molecular measures, including cystoscopy, imaging, urine cytology, histopathological features, established molecular markers or clinical risk models.

Clinical translation will require prespecified assays, a defined intended use, blinded analysis, independent validation and transparent reporting of analytical performance. Longitudinal bladder cancer studies should also prespecify sampling schedules, treatment exposure, progression criteria and decision thresholds. For urinary EV studies, standardised collection, stabilisation and normalisation protocols are essential before EV trajectories can be interpreted as treatment-response or resistance-monitoring tools^[25,35]^. EV-directed therapeutic strategies will additionally require selective target engagement, pharmacodynamic evidence, safety evaluation and demonstration of clinical benefit beyond standard therapies.

Ultimately, progress in bladder cancer EV-based resistance research will depend on harmonised preanalytical protocols, standardised normalisation, improved EV-subtype resolution, physiologically relevant models and prospective validation. These measures are necessary to distinguish reproducible EV signals of drug adaptation from platform-specific artefacts, treatment-induced inflammation, tissue injury and nonspecific consequences of therapy.

## FUTURE DIRECTIONS: DRUG-SPECIFIC EV STUDIES IN BLADDER CANCER

Advancing EV research in bladder cancer drug resistance will require a shift from descriptive cargo profiling to drug-specific functional studies. The central question is not simply which molecules are detectable in EV preparations but whether defined EV populations alter responses to gemcitabine- and platinum-based chemotherapy, BCG therapy and immune-checkpoint blockade. This requires linking EV source, cargo, recipient-cell response and treatment outcome within the same experimental or clinical framework.

### Defining EV populations that alter drug response

Future studies should identify which EV populations are functionally relevant in different bladder cancer treatment contexts. For gemcitabine- and platinum-based chemotherapy, studies should determine whether EVs from drug-exposed or drug-resistant bladder cancer cells alter drug sensitivity, DNA damage tolerance, apoptosis, clonogenic recovery or survival of recipient stem-like cells. For BCG therapy and immune-checkpoint blockade, studies should determine whether tumour-, immune-, or stromal-derived EVs influence antitumour immune activation and immune escape.

Molecular profiling alone is insufficient to establish functional relevance. Candidate EV populations should be tested through cargo- or subtype-specific perturbation, recipient-cell mapping and functional rescue. These approaches should follow established EV reporting and functional validation principles, including adequate EV characterisation, assessment of functional cargo transfer and careful discrimination between vesicular and non-vesicular effects^[29,32,33]^. For chemotherapy, EV cargo should be linked directly to gemcitabine or platinum responses. For BCG, the effects of EVs should be evaluated in relation to dendritic-cell activation, T-cell proliferation, macrophage polarisation, antigen presentation and checkpoint signalling. For immune-checkpoint blockade, EV-associated immune regulators should be tested for their ability to predict treatment response or modify treatment sensitivity beyond information provided by soluble biomarkers and tissue immune infiltration.

### Linking EV communication to treatment adaptation

Bladder cancer resistance may arise through both stable resistant clones and reversible treatment-tolerant states. EV-mediated communication could help explain how adaptive phenotypes are maintained or propagated across heterogeneous tumour cell populations and between tumour and microenvironmental compartments. This is particularly relevant for chemotherapy-exposed EVs that support stem-like cell survival, BCG-induced EVs that promote immune activation and EV-associated immune or stromal signals that may accompany immune escape^[3,19,22]^.

Future studies should integrate EV production, uptake and functional effects with longitudinal tumour and biofluid sampling. This would help determine whether EV changes precede resistance, accompany treatment responses or reflect inflammation, haematuria, infection or tissue injury. In bladder cancer, this distinction is especially important because urinary EVs are readily accessible but biologically heterogeneous.

### Developing actionable EV monitoring strategies

Prospective studies should determine whether serial urinary or circulating EV profiles provide information beyond cystoscopy, imaging, urine cytology, circulating tumour DNA, histopathological features and established clinical risk models. For chemotherapy, EV trajectories that may signal gemcitabine or platinum adaptation before relapse should be prioritised. For BCG-treated NMIBC, studies should test whether early urinary EV changes can distinguish effective BCG-induced immune activation from emerging BCG-unresponsive immune escape. For immune-checkpoint blockade, EV-bound immune molecules should be evaluated alongside soluble biomarkers and tumour immune features to determine whether they improve prediction of treatment response or resistance^[50]^.

EV-directed intervention should be approached cautiously. Non-selective inhibition of EV release may be undesirable because EV pathways also contribute to physiological intercellular communication and immune regulation. Future therapeutic strategies should instead focus on functionally defined EV populations, cargo-loading mechanisms, surface-recognition pathways or recipient-cell uptake processes. Such approaches should demonstrate selective target engagement and be tested in combination with standard bladder cancer therapies, including gemcitabine- and platinum-based chemotherapy, BCG therapy and immune-checkpoint blockade.

### Unresolved questions

Several unresolved questions should guide the next phase of bladder cancer EV resistance research:

1. Which therapy-induced EV subpopulations directly modify treatment response, and which primarily reflect donor-cell stress, inflammation or tissue injury across gemcitabine, platinum-based chemotherapy, BCG therapy and immune-checkpoint blockade?

2. Which EV cargoes, including proteins, non-coding RNAs, chromatin-associated molecules and immune-regulatory proteins, are functionally required for altered drug sensitivity, treatment-tolerant or stem-like states, immune activation or immune escape?

3. Can longitudinal urinary or circulating EV trajectories detect clinically relevant treatment adaptation before recurrence, progression or systemic treatment failure, while providing incremental value beyond cystoscopy, imaging, circulating tumour DNA, urine cytology and established biomarkers?

4. Which EV cargo-loading, vesicle-release, surface-recognition or recipient-cell uptake mechanisms can be selectively targeted without disrupting physiological EV-mediated functions?

Addressing these questions will clarify when EVs function as causal mediators, permissive amplifiers or biomarkers of bladder cancer drug adaptation, and will support the development of drug-specific EV models for chemotherapy resistance, BCG failure and resistance to immune-checkpoint blockade.

## CONCLUSIONS

In bladder cancer, therapeutic resistance arises through interactions among tumour cells, immune cells, stromal compartments and treatment-induced inflammatory states. EVs may contribute to this process by transmitting or reinforcing signals associated with chemotherapy tolerance, BCG-induced immune responses, immune escape and tumour–microenvironment remodelling. However, EV-associated findings should not be uniformly interpreted as direct resistance mechanisms. Their biological significance depends on the therapeutic context, cellular origin, vesicle subtype, cargo composition and recipient-cell state.

In this review, we propose an evidence-stratified framework for evaluating EVs in bladder cancer drug adaptation. Current evidence supports a spectrum of EV functions, ranging from causal mediation of drug resistance to permissive amplification of adaptive tumour–microenvironment states and biomarker associations with treatment response and progression. Currently, the strongest evidence specific to bladder cancer relates to chemotherapy-associated EV remodelling; however, the potential roles of EVs in BCG failure and immune-checkpoint blockade remain biologically plausible but are less directly established.

Future progress will require drug-specific models, subtype-resolved EV analysis, cargo-specific perturbation, functional rescue experiments and prospective longitudinal validation. The clinical value of EVs in bladder cancer will ultimately depend on whether urinary and circulating EV trajectories can distinguish treatment response, inflammation, tissue injury and emerging resistance. By linking EV biology to defined therapies and validation standards, EV research may move beyond descriptive biomarker discovery towards clinically informative monitoring and, potentially, earlier identification of bladder cancer drug resistance.

## DECLARATIONS

### AI and AI-assisted tools statement

During the preparation of this manuscript, the AI tool ChatGPT (OpenAI; GPT-5.5 Thinking; accessed 2026-09-17) was used solely for language editing and editorial refinement. The tool did not influence the study design, data collection, analysis, interpretation, or the scientific content of the work. All authors take full responsibility for the accuracy, integrity, and final content of the manuscript.

### Copyright

© The Author(s) 2026.
